# Supplementary material for: Heat adaptation of phage T7 under an extended genetic code
Source: Virus Evol. 2021 Dec 1;7(2):veab100. doi: 10.1093/ve/veab100 (PMC8923235; doi:10.1093/ve/veab100)
Supplement: veab100_Supp [file veab100_supp.zip › Sequencing_Analysis.zip!/Sequencing_Analysis/S2/evidence/JC_SIDE_1_68.html]

BRESEQ :: Evidence


| New junction evidence | | | | | | | | | | | |
| --- | --- | --- | --- | --- | --- | --- | --- | --- | --- | --- | --- |
|  | | seq id | position | reads (cov) | reads (cov) | score | skew | freq | annotation | gene | product |
| \* | ? | NC\_001604.1 | = 16604 | 3197 (0.600) | 1690 (0.320) | 335/590 | 1.7 | 34.5% | intergenic (–/–) | *–/–* | –/– |
|  | ? | NC\_001604.1 | 17028 = | 3275 (0.630) |  |  |  |  | intergenic (–/–) | *–/–* | –/– |

Only 100 of 5117 total aligned reads displayed.  

`GGGTACATGATGAAATCCAAGTAGGCTGCCGTACCGAAGAGATTGCTCAGGTGGTCATTGAGACCGCACAAGAAGCGATGCGCTGGGTTGGAGACCACTGGAACTTCCGGTGTCTTCTGGATACCGAAGGTAAGATGGGTCCTAATTGGGCGATTTGCCACTGATACAGGAGGCTACTCATGAACGAAAGACACTTAACAGGTGCTGCTTCTGAAATGCTAGTAGCCTACAAATTTACCAAAGCTGGGTACACTGTCTATTACCCTATGCTGACTCAGAGTAAAGAGGACTTGGTTGTATGTAAGGATGGTAAATTTAGTAAGGTTCAGGTTAAAACAGCCACAACGGTTCAAACCAACACAGGAGATGCCAAGCAGGTTAGGCTAGGTGGATGCGGTAGGTCCGAATATAAGGATGGAGACTTTGACATTCTTGCGGTTGTGGTTGACGAAGATGTGCTTATTTTCACATGGGACGAAGTAAAAGGTAAGACATCCATGTGTGTCGGCAAGAGAAACAAAGGCATAAAACTATAGGAGAAATTATTATGGCTATGACAAAGAAATTTAAAGTGTCCTTCGACGTTACCGCAAAGATGTCG  >  NC_001604.1/16304‑16904`  

                                                                                                                                                                                                                                                                                                            `|`                                                                                                                                                                                                                                                                                                              
 `ggTACATGATGAAATCCAAGTAGGCTGCCGTACCGAAGAGATTGCTCAGGTGGTCATTGAGACCGCACAAGAAGCGATGCGCTGGGTTGGAGACCACTGGAACTTCCGGTGTCTTCTGGATACCGAAGGTAAGATGGGTCCTAATTGGGCGATTTGCCACTGATACAGGAGGCTACTCATGAACGAAAGACACTTAACAGGTGCTGCTTCTGAAATGCTAGTAGCCTACAAATTTACCAAAGCTGGGTACACTGTCTATTACCCTATGCTGACTCAGAGTCAAGAGGACTTGGTtgtatgt                                                                                                                                                                                                                                                                                                             >  1:177774/1‑301 (MQ=255)`  
  `gTACATGATGAAATCCAAGTAGGCTGCCGTACCGAAGAGATTGCTCAGGTGGTCATTGAGACCGCACAAGAAGCGATGCGCTGGGTTGGAGACCACTGGAACTTCCGGTGTCTTCTGGATACCGAAGGTAAGATGGGTCCTAATTTGGCGATTTGCCACTGATACAGGAGGCTACTCATGAACGAAAGACACTTAACAGGTGCTGCTTCTGAAATGCTAGTAGCCTACAAATTTACCAAAGCTGGGTACACTGTCTATTACCCTATGCTGACTCAGAGTAAAGAGGACTTGGTTGTATGga                                                                                                                                                                                                                                                                                                            >  1:564702‑M1/1‑299 (MQ=255)`  
                  `ggtataagagacagaCCGAAGAGATTGCTAAGGTGGTCATGGAGACCGAACAAGAAGCGATGCGCTGGGTTGGAGACCACTGGAACTTCCGGTGTCTTCTGGATACCGAAGGTAAGATGGGTCCTAATTGGGCGATTTGCCACTGATACAGGAGGCTACTCATGAACGAAAGACACGTAACAGGTGCTGCTTCTGAAATGCTAGTAGCCTACAAAGTTACCAAAGCTGGGTACACTGTCTATTACCCTATGCTGACTCAGAGTAAAGAGGACTTGGTTGTATGTAAGGATGGTAAAGTTAg                                                                                                                                                                                                                                                                                            <  1:523588/287‑1 (MQ=255)`  
                  `aaGTAGGCTGCCGTACCGAAGAGATTGCTCAGGTGGTCATTGAGACCGCACAAGAAGCGATGCGCTGGGTTGGAGACCACTGGAACTTCCGGTGTCTTCTGGATACCGAAGGTAAGATGGGTCCTAATTGGGCGATTTGCCACTGATACAGGCGGCTACTCATGAACGAAAGACACTTAACAGGTGCTGCTTCTGAAATGCTAGTAGCCTACAAATTTACCAAAGCTGGGTACACTGTCTATTACCCTATGCTGACTCAGAGTAAAGAGGACTTGGTTGTATGTAAGGATAGTAAATTTAg                                                                                                                                                                                                                                                                                            >  1:470920/1‑301 (MQ=255)`  
                             `cctacCGAAGATATTGCTCAGGTGGTCATTGAGACCGAACAAGAAGCGAGGGGCTGGGTTGTAGACCACTGGAACTTCCGGTGTCTTCTGGATACCGAAGGTAAGAGGGGTCCTAATTGGGCGATTTGCCACTGATACAGGAGGCTACTCATGAACGAAAGACACTTAACAGGTGCTGCTTCTGAAATGCTAGTAGCCTACAAATTTACCAAAGCTGGGTACACTGTCTATTACCCTATGCTGACTCAGAGTAAAGAGGACTTGGTTGTATGgaaggattgatgacattcgtagtacgtac                                                                                                                                                                                                                                                                                 <  1:700873‑M1/299‑30 (MQ=255)`  
                             `cagaCCGAAGAGATTGCTCAGGTGGTCATTGAGACCGCACAAGAAGCGATGCGCTGGGTTGGAGACCACTGGAACTTCCGGTGTCTTCTGGATACCGAAGGTAAGATGGGTCCTAATTGGGCGATTTGCCACTGATACAGGAGGCTACTCATGAACGAAAGACACTTAACAGGTGCTGCTTCTGAAATGCTAGTAGCCTACAAATTTACCAAAGCTGGGTACACTGTCTATTACCCTATGCTGACTCAGAGTAAAGAGGACTTGGTTGTATGTAAGGATGGTAAATTTAGTAAGGTTCAgg                                                                                                                                                                                                                                                                                 <  1:251718/298‑1 (MQ=255)`  
                              `gTACCGAAGAGATTGCTCAGGTGGTCATTGAGACCGCACAAGAAGCGATGCGCTGGGTTGGAGACCACTGGAACTTCCGTTGTCTTCTGGATACCGAAGGTAAGATGGGTCCTAATTGGGCGATTTGCCACTGATACAGGAGGCTACTCATGAACGAAAGACACTTAACAGGTGCTGCTTCTGAAATGCTAGTAGCCTACAAATTTACCAAAGCTGGGTACACTGTCTATTACCCTATGCTGACTCAGAGTAAAGAGGACTTGGTTGTATGgaaggattgatgacattcgtagtacgtaca                                                                                                                                                                                                                                                                                <  1:792953‑M1/301‑31 (MQ=255)`  
                              `gTACCGAAGAGATTGCTCAGGTGGTCATTGAGACCGCACAAGAAGCGATGCGCTGGGTTGGAGACCACTGGAACTTCCGGTGTCTTCTGGATACCGAAGGTAAGATGGGTCCTAATTGGGCGATTTGCCACTGATACAGGAGGCTACTCATGAACGAAAGACACTTAACAGGTGCTGCTTCTGAAATGCTAGTAGCCTACAAATTTACCAAAGCTGGGTACACTGTCTATTACCCTATGCTGACTCAGAGTAAAGAGGACTTGGTTGTATGgaaggattgatgacattcgtagtacgtaca                                                                                                                                                                                                                                                                                >  1:506521‑M1/1‑271 (MQ=255)`  
                                 `ccGAAGAGATTGCTCAGGTGGTCATAGAGACCGCACAAGAAGCGATGCGCTGGGTTGGAGACCACTGGAACTTCCGGTGTCTTCTGGATACCGAAGGTAAGATGGGTCCTAATTGGGCGATTTGCCACTGATACAGGAGGCTACTCATGAACGAAAGACACTTAACAGGTGCTGCTTCTGAAATGCTAGTAGCCTACAAATTTACCAAAGCTGGGTACACTGTCTATTACCCTATGCTGACTCAGAGTAAAGAGGACTTGGTTGTATGgaaggattgatgacattcgtagtacgtacatca                                                                                                                                                                                                                                                                             <  1:717574‑M1/301‑34 (MQ=255)`  
                                  `agagacagttgctcaggtGGACATTGAGACCGCACAAGAAGCGATGCGCTGGGTTGGAGACCACTGGAACTTCCGGTGTCTTCTGGATACCGAAGGTAAGATGGGTCCTAATTGGGCGATTTGCCACTGATACAGGAGGCTACTCATGAACGAAAGACACTTAACAGGTGCTGCTTCTGAAATGCTAGTAGCCTACAAATTTACCAAAGCTGGGTACACTGTCTATTACCCTATGCTGACTCAGAGTAAAGAGGACTTGGTTGTATGgaaggattgatgacattcgtagtacgtacatcat                                                                                                                                                                                                                                                                            <  1:129840‑M1/293‑35 (MQ=255)`  
                                    `aaGAGATTGCTCAGGTGGTCATTGAGACCGCACAAGAAGCGATGCGCTGGGTTGGAGACCACTGGAACTTCCGGTGTCTTCTGGATACCGAAGGTAAGATGGGTCCTAATTGGGCGATTTGCCACTGATACAGGAGGCTACTCATGAACGAAAGACACTTAACAGGTGCTGCTTCTGAAATGCTAGTAGCCTACAAATTTACCAAAGCTGGATACACTGTCTATTACCCTATGCTGACTCAGAGTAAAGAGGACTTGGTTGTATGTAAGGATGGTAAATTTAGTAAGGTTCAGGTTAAAAc                                                                                                                                                                                                                                                                          <  1:107461/301‑1 (MQ=255)`  
                                        `gaTTGCTCAGGTGGTCATTGAGACCGCACAAGAAGCGATGCGCTGGGTTGGAGACCACTGGAACTTCCGGTGTCTTCTGGATACCGAAGGTAAGATGGGTCCTAATTGGGCGATTTGCCACTGATACAGGAGGCTACTCATGAACGAAAGACACTTAACAGGTGCTGCTTCTGAAATGCTAGTAGCCTACAAATTTACCAAAGCTGGGTACACTGTCTATTACCCTATGCTGACTCAGAGTAAAGAGGACTTGGTTGTATGgaaggattgatgacattcgtagtacgtctgtctcttatac                                                                                                                                                                                                                                                                      >  1:807815‑M1/1‑261 (MQ=255)`  
                                        `gaTTGCTCAGGTGGTCATTGAGACCGCACAAGAAGCGATGCGCTGGGTTGGAGACCACTGGAACTTCCGGTGTCTTCTGGATACCGAAGGTAAGATGGGTCCTAATTGGGCGATTTGCCACTGATACAGGAGGCTACTCATGAACGAAAGACACTTAACAGGTGCTGCTTCTGAAATGCTAGTAGCCTACAAATTTACCAAAGCTGGGTACACTGTCTATTACCCTATGCTGACTCAGAGTAAAGAGGACTTGGTTGTATGTAAGGATGGTAAATTTAGTAAgctgtctcttatacacatt                                                                                                                                                                                                                                                                      >  1:83999/1‑283 (MQ=255)`  
                                                 `ggtggtCATTGAGACCGCACAAGAAGCGATGCGCTGGGTTGGAGACCACTGGAACTTCCGGTGTCTTCTGGATACCGAAGGTAAGATGGGTCCTAATTGGGCGATTTGCCACTGATAAAGGAGGCTACTCATGAACGAAAGAAACTTAACAGGTGCTGCTTCTGAAATGCTAGTAGCCTACAAATTTACCAAAGCTGGGTACAATGTCTATTCCCCTATGCTGACTCAGAGTAAAGCGGACTTGGTTGTATGTACGGATGGTAAATTTAGTAAGGATCAGGTTAAAATAGCCACaaagaat                                                                                                                                                                                                                                                             >  1:155161/1‑296 (MQ=255)`  
                                                     `gtCATTGAGACCGCACAAGAAGCGATGCGCTGGGTTGGAGACCACTGGAACTTCCGGTGTCTTCTGGATACCGAAGGTAAGATGGGTCCTAATTGGGCGATTTGCCACTGATACAGGAGGCTACTCATGAACGAAAGACACTTAACAGGTGCTGCTTCTGAAATGCTAGTAGCCTACAAATTTACCAAAGCTGGGTACACTGTCTATTACCCTATGCTGACTCAGAGTAAAGAGGACTTGGTTGTATGgaaggattgatgacattcgtagtacgtacatcatttcgtgaggccattaagga                                                                                                                                                                                                                                                         >  1:170046‑M1/1‑248 (MQ=255)`  
                                                       `cATTGAGACCGCACAAGAAGCGATGCGCTGGGTTGGAGACCACTGGAACTTCCGGTGTCTTCTGGATACCGAAGGTAAGATGGGTCCTAATTGGGCGATTTGCCACTGATACAGGAGGCTACTCATGAACGAAAGACACTTAACAGGTGCTGCTTCTGAAATGCTAGTAGCCTACAAATTTACCAAAGCTGGGTACACTGTCTATTACCCTATGCTGACTCAGAGTAAAGAGGACTTGGTTGTATGTAAGGATGGTAAATTTAGTAAGGTTCAGGTTAAAACAGCCACAACTGtctcttat                                                                                                                                                                                                                                                       >  1:305784/1‑294 (MQ=255)`  
                                                            `agaCCGCACAAGAAGCGATGCGCTGGGTTGGAGAACACTGGAACTTCCGGTGTCGTCTGGATACCGAAGGTAAGATGGGTCCTAATAGGGCGATTTGCCACTGATACAGGAGGCTACTCATGAACGAAAGACACTTAACAGGTGCTGCTTCTGAAATGCTAGTAGCCTACAAATTTACCAAAGCTGGGTACACTGTCTATTACCCTATGCTGACTCAGAGTAAAGAGGACTTGGTTGTATGTAAGGATGGTAAATTTAGTAAGGTTCAGGTTAAAACAGCCACAACGGTTCAAACCGacac                                                                                                                                                                                                                                                  <  1:785151/301‑1 (MQ=255)`  
                                                                 `gCACAAGAAGCGATGCGCTGGGTTGGAGACCACTGGAACTTCCGGTGTCTTCTGGATACCGAAGGTAAGATGGGTCCTAATTGGGCGATTTGCCACTGATACAGGAGGATACTCATGAACGAAAGACACTTAACAGGTGCTGCTTCTGAAATGCTAGTAGCCTACAAATTTACCAAAGCTGGGTACACTGTCTATTACCCTATGCTGACTCAGAGTAAAGAGGACTTGGTTGTATGTAAGGATGGTAAATTTAGTAAGGTTCAGGTTAAAACAGCCACAACGGTTCAAACCAACACAGgag                                                                                                                                                                                                                                             <  1:147834/301‑1 (MQ=255)`  
                                                                       `gtgtataagagaaaagttggagaccactggaACTTCCGGTGTCTTCTGGATACCGAAGGTAAGATGGGTCCTAATTGGGAGATTTGCCACTGATACAGGAGGCTACTCATGAACGAAAGACACTTAACAGGTGCTGCTTCTGAAATGCTAGTAGCCTACAAATTTACCAAAGCTGGGTACACTGTCTATTACCCTATGCTGACTCAGAGTAAAGAGGACTTGGTTGTATGgaaggattgatgacattcgtagtacgtacatcatttcgtgaggccattaaggacatgcacgaagagtatgc                                                                                                                                                                                                                                       <  1:725304‑M1/286‑72 (MQ=255)`  
                                                                               `gcgcTGGGTTGGAGACCACTGGAACTTCCGGTGTCTTCTGGATACCGAAGGTAAGATGGGTCCTAATTGGGCGATTTGCCACTGATACAGGAGGCTACTCATGAACGAAAGACACTTAACAGGTGCTGCTTCTGAAATGCTAGTAGCCTACAAATTTACCAAAGCTGGGTACACTGTCTATTACCCTATGCTGACTCAGAGTAAAGAGGACTTGGTTGTATGgaaggattgatgacattcgtagtacgtacatcatttcgtgaggccattaaggacatgcacgaagagtatgcagactgtc                                                                                                                                                                                                                               >  1:6267‑M1/1‑222 (MQ=255)`  
                                                                                    `gggTTGGAGACCACTGGAACTTCCGGTGTCTTCTGGATACCGAAGGTAAGATGGGTCCTAATTGGGCGATTTGCCACTGATACAGGAGGCTACTCATGAACGAAAGACACTTAACAGGTGCTGCTTCTGAAATGCTAGTAGCCTACAAATTTACCAAAGCTGGGTACACTGTCTATTACCCTATGCTGACTCAGAGTAAAGAGGACTTGGTTGTATGgaaggattgatgacattcgtagtacgtacatcatttcgtgaggccattaaggacatgcacgaagagtatgcagataaggactct                                                                                                                                                                                                                          >  1:358622‑M1/1‑217 (MQ=255)`  
                                                                                    `gggTTGGAGACCACTGGAACTTCCGGTGTCTTCTGGATACCGAAGGTAAGATGGGTCCTAATTGGGCGATTTGCCACTGATACAGGAGGCTACTCATGAACGAAAGACACTTAACAGGTGCTGCTTCTGAAATGCTAGTAGCCTACAAATTTACCAAAGCTGGGTACACTGTCTATTACCCTATGCTGACTCAGAGTAAAGAGGACTTGGTTGTATGTAAGGATGGTAAATTTAGTAAGGTTCAGGTTAAAACAGCCACAACGGTTCAAACCAACACAGGAGATGCCAAGCAGGTTAGGCt                                                                                                                                                                                                                          <  1:234957/301‑1 (MQ=255)`  
                                                                                    `gggTTGGAGACCACTGGAACTTCCGGTGTCTTCTGGATACCGAAGGTAAGATGGGTCCTAATTGGGCGATTTGCCACTGATACAGGAGGCTACTCATGAACGAAAGACACTTAACAGGTGCTGCTTCTGAAATGCTAGTAGCCTACAAATTTACCAAAGCTGGGTACACTGTCTATTACCCTATGCTGACTCAGAGTAAAGAGGACTTGGTTGTATGTAAGGATGGTAAATTTAGTAAGGTTCAGGTTAAAACAGCCACAACGGTTCAAACCAACACAGGAGATGCCAAGCAGGTTAGGCt                                                                                                                                                                                                                          <  1:205463/301‑1 (MQ=255)`  
                                                                                    `gggTTGGAGACCACTGGAACTTCCGGTGTCTTCTGGATACCGAAGGTAAGATGGGTCCTAATTGGGCGATTTGCCACTGATACAGGAGGCTACTCATGAACGAAAGACACTTAACAGGTGCTGCTTCTGAAATGCTAGTAGCCTACAAATTTACCAAAGCTGGGTACACTGTCTATTACCCTATGCTGACTCAGAGTAAAGAGGACTTGGTTGTATGTAAGGATGGTAAATTTAGTAAGGTTCAGGTTAAAACAGCCACAACGGTTCAAACCAACACAGGAGATGCCAAGCAGGTTAGGCt                                                                                                                                                                                                                          <  1:709211/301‑1 (MQ=255)`  
                                                                                    `gggTTGGAGACCACTGGAACTTCCGGTGTCTTCTGGATACCGAAGGTAAGATGGGTCCTAATTGGGCGATTTGCCACTGATACAGGAGGCTACTCATGAACGAAAGACACTTAACAGGTGCTGCTTCTGAAATGCTAGTAGCCTACAAATTTACCAAAGATGGGTACACTGTCTATTACCCTATGCTGACTCAGAGTAAAGAGGACTTGGTTGTATGTAAGGATGGTAAATTTAGTAAGGTTCAGGTTAAAACAGCCACAACGGTTCAAACCTACACAGGAGATGccctgtctcttataca                                                                                                                                                                                                                          >  1:799198/1‑287 (MQ=255)`  
                                                                                       `tgtgtataagagacagcttccggtgtcttccggATACCGAAGGTAAGATGGGTCCTAATTGGGCGATTTGCCACTGATACAGGAGGCTACTCATGAACGAAAGACACTTAACAGGTGCTGCTTCTGAAATGCTAGTAGCCTACAAATTTACCAAAGCTGGGTACACTGTCTATTACCCTATGCTGACTCAGAGTAAAGAGGACTTGGTTGTATGgaaggattgatgacattcgtagtacgtacatcatttcgtgaggccattaaggacatgcacgaagagtatgcagataaggactctttc                                                                                                                                                                                                                       <  1:431162‑M1/285‑88 (MQ=255)`  
                                                                                         `ggAGACCACTGGAACTTCCGGTGTCTTCTGGATACCGAAGGTAAGATGGGTCCTAATTGGGCGATTTGCCACTGATACAGGAGGCTACTCATGAACGAAAGACACTTAACAGGTGCTGCTTCTGAAATGCTAGTAGCCTACAAATTTACCAAAGCTGGGTACACTGTCTATTACCCTATGCTGACTCAGAGTAAAGAGGACTTGGTTGTATGgaaggattgatgacattcgtagtacgtacatcatttcgtgaggccattaaggacatgcacgaagagtatgcctgtctcttatacacatt                                                                                                                                                                                                                     >  1:65254‑M1/1‑212 (MQ=255)`  
                                                                                                 `cTGGAACTTCCGGTGTCTTCTGGATACCGAAGGTAAGATGGGTCCTAATTGGGCGATTTGCCACTGATACAGGAGGCTACTCATGAACGAAAGACACTTAACAGGTGCTGCTTCTGAAATGCTAGTAGCCTACAAATTTACCAAAGCTGGGTACACTGTCTATTACCCTATGCTGACTCAGAGTAAAGATGACTTGGTTGTATGTAAGGATGGTAAATTTAGTAAGGTTCAGGTTAAAACAGCCACAACGGTTCAAACCAACACAGGAGATGCCAAGCAGGTTAGGCTAGGTGGATGCGGt                                                                                                                                                                                                             <  1:464100/301‑1 (MQ=255)`  
                                                                                                               `gtCTTCTGGATACCGAAGGTAAGATGGGTCCTAATTGGGCGATTTGCCACTGATACAGGAGGCTACTCATGAACGAAAGACACTTAACAGGTGCTGCTTCTGAAATGCTAGTAGCCTACAAATTTACCAAAGCTGGGTACACTGTCTATTACCCTATGCTGACTCAGAGTAAAGAGGACTTTGTTGTATGTAAGGATGGTAAATTTAGTAAGGTTCAGGTTAAAACAGCCACAACGGTTCAAACCAACACAGGAGATGCCAAGCAGGTTAGGCTAGGTGGATGCGGTAGGTCCGAATATaa                                                                                                                                                                                               >  1:243642/1‑301 (MQ=255)`  
                                                                                                                 `cttctGGATACCGAAGGTAAGATGGGTCCTAATTGGGCGATTTGCCACTGATACAGGAGGCTACTCATGAACGAAATACACTTAACAGGTGCTGCTTCTGAAATGCTAGTAGCCTACAAATTTACCACAGCTGGGTACACTGTCTATTACCCTATGCTGACTCTGAGTAAAGAGGACTTGGTTGTATGTAAGGATGGTAAATTTAGTAAGTTTCAGGTTAAAACAGCCACAACGGTTCAAACCAACACAGGAGATGCCAAGCAGGTTAGGCtctgtctcttatacacatctccgagcccac                                                                                                                                                                                             >  1:288689/1‑272 (MQ=255)`  
                                                                                                                 `cttctGGATACCGAAGGTAAGATGGGTCCTAATTGGGCGATTTGCCACTGATACAGGAGGCTACTCATGAACGAAAGACACTTAACAGGTGCTGCTTCTGAAATGCTAGTAGCCTACAAATTTACCAAAGCTGGGTACACTGTCTATTACCCTATGCTGACTCAGAGTAAAGAGGACTTGGTTGTATGgaaggattgatgacattcgtagtacgtacatcatttcgtgaggccattaaggacatgcacgaagagtatgcagataaggactctttcaaacaatctcctgcaacagtacgggg                                                                                                                                                                                             >  1:570009‑M1/1‑188 (MQ=255)`  
                                                                                                                  `ttctGGATACCGAAGGTAAGATGGGTCCTAATTGGGCGATTTGCCACTGATACAGGAGGCTACTCATGAACGAAAGACACTTAACAGGTGCTGCTTCTGAAATGCTAGTAGCCTACAAATTTACCAAAGCTGGGTACACTGTCTATTACCCTATGCTGACTCAGAGTAAAGAGGACTTGGTTGTATGTAAGGATGGTAAATTTAGTAAGGTTCAGGTTAAAACAGCCACAACGGTTCAAACCAACACAGGAGATGCCAAGCAGGTTAGGCTAGGTGGATGCGGTAGGTCCGAATATAAGGa                                                                                                                                                                                            <  1:739984/301‑1 (MQ=255)`  
                                                                                                                   `tctGGATACCGAAGGTAAGATGGGTCCTAATTGGGCGATTTGCCACTGATACAGGAGGCTACTCATGAACGAAAGACACTTAACAGGTGCTGCTTCTGAAATGCTAGTAGCCTACAAATTTACCAAAGCTGGGTACACTGTCTATTACCCTATGCTGACTCAGAGTAAAGAGGACTTGGTTGTATGgaaggattgatgacattcgtagtacgtacatcatttcgtgaggccattaaggaaatgcacgaagagtatgcagataaggactctttcaaacaatctcctgcaacagtacgggagg                                                                                                                                                                                           >  1:531766‑M1/1‑186 (MQ=255)`  
                                                                                                                       `gATACCGAAGGTAAGATGGGTCCTAATTGGGCGATTTGCCACTGATACAGGAGGCTACTCATGAACGAAAGACACTTAACAGGTGCTGCTTCTGAAATGCTAGTAGCCTACAAATTTACCAAAGCTGGGTACACTGTCTATTACCCTATGCTGACTCAGAGTAAAGAGGACTTGGTTGTATGTAAGGATGGTAAATTTAGTAAGGTTCAGGTTAAAACAGCCACAACGGTTCAAACCAACACAGGAGATGCCAAGCAGGTTAGGCTAGGTGGATGCGGTAGGTCCGAATATAAGGATGgag                                                                                                                                                                                       <  1:13942/301‑1 (MQ=255)`  
                                                                                                                           `ccGAAGGTAAGATGGGTCCTAATTGGGCGAGTTGCCACTGATACAGGAGGCTACTCATGAACGAAAGACACTTAACAGGTGCTGCTTCTGAAATGCTAGTAGCCTACAAATTTACCAAAGCTGGGTACACTGTCTATTACCCTATGCTGACTCAGAGTAAAGAGGACTTGGTTGTATGgaaggattgatgacattcgtagtacgtacatcatttcgtgaggccattaaggacatgcacgaagagtatgcagataaggactctttcaaacaatctcctgcaacagtacgggaggtgttctga                                                                                                                                                                                   <  1:455235‑M1/301‑124 (MQ=255)`  
                                                                                                                              `aaGGTAAGATGGGTCCTAATTGGGCGATTTGCCACTGATACAGGAGGCTACTCATGAACGAAAGACACTTAACAGGTGCTGCTTCTGAAATGCTAGTAGCCTACAAATTTACCCACGCTGGGTCCACTGTCTATTACCCTATGCTGACTCAGCGTAAAGAGGACTTGGATGTATGTAAGGATGGTAAATTTAGTAAGGTTCACGTTAACACAGCCACAACGGTTCAAACCAACACAGGAGATGCCAAGCAGGTTCAGCTAGGTGGATGCGGTAGGTCCGAATATAAGGATGGAGACTTTGa                                                                                                                                                                                >  1:198192/1‑301 (MQ=255)`  
                                                                                                                               `aGGTAATATGGGTCCTAATTGGGCGATTTGCCACTGATACAGGAGGCTACTCATGAACGAAAGACACTTAACAGGTGCTGCTTCTGAAATGCTAGTAGCCTACAAATTTACCAAAGCTGGGTACACTGTCTATTACCCTATGCTGACTTAGACTCAAGAGGACTTGGTTGTAAGTAAGGATGGTAAAATTATTAAGGATCAGGTTAAAACAGCCACAACGGTTAAAACCAACACAGGAGATGACAAGCCGGATAGGTTAGGGGGATGCGGTAGGTCCGAATATAAGTGTAGAGACTTCGAc                                                                                                                                                                               >  1:54287/1‑301 (MQ=255)`  
                                                                                                                               `aGGTAAGATGGGTCCTAATTGGGCGATTTGCCACTGATACAGGAGGCTACTCATGAACGAAAGACACTTAACAGGTGCTGCTTCTGAAATGCTAGTAGCCTACAAATTTACCAAAGCTGGGTACACTGTCTATTACCCTATGCTGACTCAGAGTAAAGAGGACTTGGTTGTATGTAAGGATGGTAAATTTAGTAAGGTTCAGGTTAAAACAGCCACAACGGTTCAAACCAACACAGGAGATGCCAAGCAGGTTAGGCTAGGTGGATGCGGTAGGTCCGAATATAAGGATGGAGACTTTGAc                                                                                                                                                                               <  1:281167/301‑1 (MQ=255)`  
                                                                                                                                `ggTAAGATGGGTCCTAATTGGGCGATTTGCCACTGATACATGAGGCTACTCATGAACGAAAGACACTTAACAGGTGCTGCTTCTGAAATGCTAGTAGCCTACAAATTTACCAAAGCTGGGTACACTGTCTATTACCCTATGCTGACTCAGAGTAAAGAGGACTTGGTTGTATGTAAGGATGGTAAATTTAGTAAGGTTCAGGTTAAAACAGCCACAACGGTTCAAACCAACACAGGAGATGCCAAGCAGGTTAGGCTAGGTGGATGCGGTAGGTCCGAatctgtctcttatacacatctcc                                                                                                                                                                              >  1:693257/1‑280 (MQ=255)`  
                                                                                                                                     `gATGGGTCCTAATTGGGCGATTTGCCACTGATACAGGAGGCTACTCATGAACGAAAGACACTTAACAGGTGCTGCTTCTGAAATGCTAGTAGCCTACAAATTTACCAAAGCTGGGTACACTGTCTATTACCCTATGCTGACTCAGAGTAAAGAGGACTTGGTTGTTTGgaaggattgatgacattcgtagtacgtacatcatttcgtgaggccattaaggacatgcactaagagtatgcagataaggactctttcaaacaatctcctgcaacagtacgggaggtgttctgatgtctgacta                                                                                                                                                                         >  1:747733‑M1/1‑168 (MQ=255)`  
                                                                                                                                         `ggTCCTAATTGGGCGATTTGCCACTGATACAGGAGGCTACTCATGAACGAAAGACACTTAACAGGTGCTGCTTCTGAAATGCTAGTAGCCTACAAATTTACCAAAGCTGGGTACACTGTCTATTACCCTATGCTGACTCAGAGTAAAGAGGACTTGGTTGTATGgaaggattgatgacattcgtagtacgtacatcatttcgtgaggccattaaggacatgcacgaagagtatgcagataaggactctttcaaacaatctcctgcaacagtacgggaggtgttctgatgtctgactacctg                                                                                                                                                                     >  1:646159‑M1/1‑164 (MQ=255)`  
                                                                                                                                               `aaTTGGGCGATTTGCCACTGATACAGGAGGCTACTCATGAACGAAAGACACTTAACAGGTGCTGCTTCTGAAATGCTAGTAGCCTACAAATTTACCAAAGCTGGGTACACTGTCTATTACCCTATGCTGACTCAGAGTAAAGAGGACTTGGTTGTATGTAAGGATGGTAAATTTAGTAAGGTTCAGGTTAAAACAGCCACAACGGTTCAAACCAACACAGGAGATGCCAAGCAGGTTAGGCTAGGTGGATGCGGTAGGTCCGAATATAAGGATGGAGACTTTGACATTCTTGCGGTtgctt                                                                                                                                                               >  1:824213/1‑298 (MQ=255)`  
                                                                                                                                                             `ccACTGATACAGGAGGCTACTCATGAACGAAAGACACTTAACAGGTGCTGCTTCTGAAATGCTAGTAGCCTACAAATTTACCAAAGCTGGGTACACTGTCTATTACCCTATGCTGACTCAGAGTAAAGAGGACTTGGTTGTATGgaaggattgatgacattcgtagtacgtacatcatttcgtgaggccattaaggacatgcacgaagagtatgcagataaggactctttcaaacaatctcctgcaacagtacgggaggtgttctgatgtctgactacctgaaagtgctgcaagcctgtct                                                                                                                                                 >  1:49988‑M1/1‑144 (MQ=255)`  
                                                                                                                                                                `cTGATACAGGAGGCTACTCATGAACGAAAGACACTTAACAGGTGCTGCTTCTGAAATGCTAGTAGCCTACAAATTTACCAAAGCTGGGTACACTGTCTATTACCCTATGCTGACTCAGAGTAAAGAGGACTTGGTTGTATGTAAGGATGGTAAATTTAGTAAGGTTCAGGTTAAAACAGCCACAACGGTTCAAACCAACACAGGAGATGCCAAGCAGGTTAGGCTAGGTGGATGCGGTAGGTCCGAATATAAGGATGGAGACTTTGACATTCTTGCGGTTGTGGTTGACGAAGATGTGCtt                                                                                                                                              >  1:762006/1‑301 (MQ=255)`  
                                                                                                                                                                 `tGATACAGGAGGCTACTCATGAACGAAAAACGCTGAACAGGTGCTGCTTCTGAAATGCTAGTAGCCTACAAATTTACCAAATGTGGGTAGACTGTCTATTACCCTATGCTGACTCAGAGGATAGAGGACTGGGTTGTGTTgaaggattgatgacatacgtagtacgtacatcagttcgtgaggcgattaagaacatgcacgagaagtatacagataaggactctttcaaacaatctcctgcaacagtgcggcaggtgttctgatgtctgactacctgaaagtgctgcaagcaatcaaaagt                                                                                                                                             <  1:93054‑M1/301‑162 (MQ=255)`  
                                                                                                                                                                  `gATACAGGAGGCTACTCATGAACGAAAGACACTTAACAGGTGCTGCTTCTGAAATGCTAGTAGCCTACAAATTTACCAAAGCTGGGTACACTGTCTATTACCCTATGCTGACTCAGAGTAAAGAGGACTTGGTTGTATGTAAGGATGGTAAATTTAGTAAGGTTCAGGTTAAACCAGCCACAACGGTTCAAACCAACACAGGAGATGCCAAGCAGGTTAGGCTAGGTGGATGCGGTAGGTACGAATATAAGGATGGAGACTTTGACATTCTTGCGGTTGTGGTTTACGAAGactgtctctt                                                                                                                                            >  1:3974/1‑292 (MQ=255)`  
                                                                                                                                                                    `tACAGGAGGCTACTCATGAACGAAAGACACTTAACAGGTGCTGCTTCTGAAATGCTAGTAGCCTACAAATTTACCAAAGCTGGGTACACTGTCTATTACCCTATGCTGACTCAGAGTAAAGAGGACTTGGTTGTATGTACGGATGGTAAATTTAGTAAGGTTCAGGTTAAAACAGCCACAACGGTTCAAACCAACACAGGAGATGCCAAGCAGGTTAGGCTAGGTGGATGCGGTAGGTCCGAATATAAGGATGGAGACTTTGACATTCCTgtctcttatacacatctccgagcctacgaga                                                                                                                                          >  1:351213/1‑271 (MQ=255)`  
                                                                                                                                                                    `aaCAGGAGGCTACTCATGAACGAAAGACACTTAACAGGTGCTGCTTCTGAAATGCTAGTAGCCTACAAAGTTACCAAAGCTGGGGACACTGTCTATTAACCTATGCTGACTCAGAGTAAAGAGGACTTGGTTGTATGTAAGGATGGTAAATTTAGTAATGTTCAGGTTAAAACAGCCACAACGGTTCAAACCAACACAGGAGATGCCAAGCAGGTTAGGCTAGGTGGATGCGGTAGGTCCGAATATAAGGATGGAGACTTTGACATTCTTGCGGTTGTGGTTGACGAAGATGTGCTTAttt                                                                                                                                          <  1:389745/300‑1 (MQ=255)`  
                                                                                                                                                                            `gCTACTCATGAACGAAAGACACTTAACAGGTGCTGCGTCTGAAATGCTAGTAGCCTACAAATTTACCAAAGCTGGGTACACTGTCTATTACCCTATGCTGACTCAGAGTAAAGAGGACTTGGTAGTATGgaaggattgatgacattcgtagtacgtacatcatttcgtgaggccattaaggacatgcacgaagagtatgcagataaggactctttcaaacaatctcctgcaacagtacgggaggtgttctgatgtctgactacctgaaagtgctgcaagcaatcaaaagttgccctaagac                                                                                                                                  <  1:583786‑M1/301‑173 (MQ=255)`  
                                                                                                                                                                            `agatgtgtataagagacagcacttaacaggtgctgcttctgAAATGCTAGTAGCCTACAAATTTACCAAAGCTGGGTACACTGTCTATTACCCTATGCTGACTCAGAGTAAAGAGGACGTGGTTGTATGgaaggattgatgacattcgtagtacgtacatcatttcgtgaggccattaaggacatgcacgaagagtatgcagataaggactctttcaaacaatctcctgcaacagtacgggaggtgttctgatgtctgactacctgaaagtgctgcaagcaatcaaaagttgccctaagac                                                                                                                                  <  1:372015‑M1/282‑173 (MQ=255)`  
                                                                                                                                                                               `aCTCATGAACGAAAGACACTTAACAGGTGCTGCTTCTGAAATGCTAGTAGCCTACAAATTTACCAAAGCTGGGTACACTGTCTATTACCCTATGCTGACTCAGAGTAAAGAGGACTTGGTTGTATGTAAGGATGGTAAATTTAGTAAGGTTCAGGTTAAAACAGCCACAACGGTTCAAACCAACACAGGAGATGCCAAGCAGGTTAGGCTAGGTGGATGAGGTAGGTCCGAATATAAGGATGGAGACTTTGACATTCTTGCGGTTGTGGTTGACGAAGATGTGCTTATTTTCACATGGGAc                                                                                                                               >  1:217642/1‑301 (MQ=255)`  
                                                                                                                                                                                             `gACACTTAACAGGTGCTGCTTCTGAAATGCTAGTAGCCTACAAATTTACCAAAGCTGGGTACACTGTCTATTACCCTATGCTGACTCAGAGTAAAGAGGACTTGGTTGTATGTAAGGATGGTAAATTTAGTAAGGTTCAGGTTAAAACAGCCACAACGGTTCAAACCAACACAGGAGATGCCAAGCAGGTTAGGCTAGGTGGATGCGGTAGGTCCGAATATAAGGATGGAGACTTTGACATTCTTGCGGTTGTGGTTGACGAAGATGTGCTTATTTTAACATGGGACGAAGTAAAAGTTaa                                                                                                                 >  1:344846/1‑301 (MQ=255)`  
                                                                                                                                                                                                `acTTAACAGGTGCTGCTTCTGAAAGGCGTGGTTTTTATGTATGTACCAGTGCGGTGTACTTGGTCTATTACCCTATGCTGACGCAGAGTAAAGAGGACTTGGGTGTATGgaaggattgatgacattcgtagtacgtacatcatgtcgtgaggccattaaggacatgcacgaagagtatgcagataaggactctttcaaacaatctcctgcaacagtacgggaggtgttctgatgtctgactacctgaaagtgctgcaagcaatcaaaagttgccctaagactttccagtccaactatgtac                                                                                                              <  1:403975‑M1/301‑193 (MQ=255)`  
                                                                                                                                                                                                 `cTTAACAGGTGCTGCTTCTGAAATGCTAGTAGCCTACAAATTTACCAAAGCTGGGTACCCTGTCTATTACCCTATGCTGACTCAGAGTAAAGAGGACTTGGTTGTATGgaaggattgatgacattcgtagtacgtacatcatttcgtgaggccattaaggacatgcccgaagcgtatgcagataaggactctttcaaacaatctcctgcaacagtacgggaggtgttctgatgtctgactacctgaaagtgctgcaagcaatcaaaagttgccctaagactttccagtccaactatgtacg                                                                                                             >  1:41840‑M1/1‑108 (MQ=255)`  
                                                                                                                                                                                                 `cTTAACAGGTGCTGCTTCTGAAATGCTAGTAGCCTACAAATTTACCAAAGCTGGGTACACTGTCTATTACCCTATGCTGACTCAGAGTAAAGAGGACTTGGTTGTATGTAAGGATGGTAAATTTAGTAAGGTTCAGGTTAAAACAGCCACAACGGTTCAAACCAACACAGGAGATGCCAAGCAGGTTAGGCTAGGTGGATGCGGTAGGTCCGAATATAAGGATGGAGACTTTGACATTCTTGCGGTTGTGGTTGACGAAGATGTGCTTATTTTCACATGGGACGAAGTAAAAGGTAAGACa                                                                                                             <  1:536855/301‑1 (MQ=255)`  
                                                                                                                                                                                                 `cTTAACAGGTGCTGCTTCTGAAATGCTAGTAGCCTACAAATTTACCAAAGCTGGGTACACTGTCTATTACCCTATGCTGACTCAGAGTAAAGAGGACTTGGTTGTATGTAAGGATGGTAAATTTAGTAAGGTTCAGGTTAAAACAGCCACAACGGTTCAAACCAACACAGGAGATGCCAAGCAGGTTAGGCTAGGTGGATGCGGTAGGTCCGAATATAAGGATGGAGACTTTGACATTCTTGCGGTTGTGGTTGACGAAGATGTGCTTATTTTCACATGGGACGAAGTAAAAGGTAAGACa                                                                                                             >  1:313395/1‑301 (MQ=255)`  
                                                                                                                                                                                                 `cTTAACAGGTGCTGCTTCTGAAATGCTAGTAGCCTACAAATTTACCAAAGCTGGGTACACTGTCTATTACCCTATGCTGACTCAGAGTAAAGAGGACTTGGTTGTATGTAAGGATGGTAAATTTAGTAAGGTTCAGGTTAAAACAGCCACAACGGTTCAAACCAACACAGGAGATGCCAAGCAGGTTAGGCTAGGTGGATGCGATAGGTCCGAATATAAGGATGGAGACTTTGACATTCTTGCGGTTGTGGTTGACGAAGATGTGCTTATTTTCACATGGGACGAAGTAAAAGGTAAGACa                                                                                                             >  1:409021/1‑301 (MQ=255)`  
                                                                                                                                                                                                        `ggTGCTGCTTCTGAAATGCTAGTAGCCTACAAATTTACCAAAGCTGGGTACACTGTCTATTACCCTATGCTGACTCAGAGTAAAGAGGACTTGGTTGTATGTAAGGATGGTAAATTTAGTAAGGTTCAGGTTAAAACAGCCACAACGGTTC‑AACCAACACAGGAGATGCCAAGCAGGTTAGGCTAGGTGGATGCGGTAGGTCCGAATATAAGGATGGAGACTTTGACATTCTTGCGGTTGTGGTTGACGAAGATGTGCTTATTTTCACATGGGACGAAGTAAAAGGTAAGACATCCAtgtg                                                                                                     <  1:5579/301‑1 (MQ=255)`  
                                                                                                                                                                                                                 `agagacaggctagtagcCTACAAATTTACCAAAGCTGGGTACACTGTCTATTACCCTATGCTGACTCAGAGTAAAGAGGACTTGGTTGTATGgaaggattgatgacattcgtagtacgtacatcatttcgtgaggccattaaggacatgcacgaagagtatgcagataaggactctttcaaacaatctcctgcaacagtacgggaggtgttctgatgtctgactacctgaaagtgctgcaagcaatcaaaagttgccctaagactttccagtccaactatgtacggaacaatgcgagcctc                                                                                             <  1:25935‑M1/293‑210 (MQ=255)`  
                                                                                                                                                                                                                  `cTGAAATGCTAGTAGCCTACAAATTTACCAAAGCTGGGTACACTGTCTATTACCCTATGCTGACTCAGAGTAAAGAGGACTTGGTTGTATGTAAGGATGGTAAATTTAGTAAGGTTCAGTTTAAAACAGCCACAACGGTTCAAACCAACACAGGAGATGCCAAGCAGGTTAGGCTAGGTGGATGCGGTAGGTCCGAATATAAGGATGGAGACTTTGACATTCTTGCGGTTGTGGTTGACGAAGATGTGCTTATTTTCACATGGGACGAAGTAAAAGGTAAGACATCCATGTGTGTCGGCaa                                                                                            >  1:551217/1‑301 (MQ=255)`  
                                                                                                                                                                                                                     `aaaTGCTAGTAGCCTACAAATTTACCAAAGCTGGGTACACTGTCTATTACCCTATGCTGACTCAGAGTAAAGAGGACTTGGTTGTATGTAAGGATGGTAAATTTAGTAAGGTTCAGGTTAAAACAGCCACAACGGTTAAAACCAACACAGGAGATGCCAAGCAGGTTAGGCTAGGTGGATGCGGTAGGTCCGAATATAAGGATGGAGACTTTGACATTCTTGCGGTTGTGGTTGACGAAGATGTGCTTATTTTCACATGGGACGAAGTAAAAGGTAAGACATCCATGTGTGTCGGCAagag                                                                                         <  1:331208/301‑1 (MQ=255)`  
                                                                                                                                                                                                                         `gCTAGTAGCCTACAAATTTACCAAAGCTGGGTACACTGTCTATTACCCTATGCTGACTCAGAGTAAAGAGGACTTGGTTGTATGgaaggattgatgacattcgtagtacgtacatcatttcgtgaggccattaaggacatgcacgaagagtatgcagataaggactctttcaaacaatctcctgcaacagtacgggaggtgttctgatgtctgactacctgaaagtgctgcaagcaatcaaaagttgccctaagactttccagtccaactatgtacggaacaatgcgagcctcgtagcggg                                                                                     >  1:733002‑M1/1‑84 (MQ=255)`  
                                                                                                                                                                                                                           `tataatagacagaaTTTACCAAAGCTGGGTACACTGTCTATTACCCTATGCTGACTCAGAGTAAAGAGGACTTGGTTGTATGTAAGGATGGTAAATTTAGTAAGGTTCAGGTTAAAACAGCCACAACGGTTCAAACCAACACAGGAGATGCCAAGCAGGTTAGGCTAGGTGGATGCGGTAGGTCCGAATATAAGGATGGAGACTTTGACATTCTTGCGGTTGTGGTTGACGAAGATGTGCTTATTTTCACATGGGACGAAGTAAAAGGTAAGACATCCATGTGTGTCGGCAAGAGAAACaa                                                                                   <  1:477621/289‑1 (MQ=255)`  
                                                                                                                                                                                                                                              `cAAAGCTGGGTACACTGTCTATTACCCTATGCTGACTCAGAGTAAAGAGGACTTGGTTGTATGTAAGGATGGTAAATTTAGTAAGGTTCAGGTTAAAACAGCCACAACGGTTCAAACCAACACAGGAGATGCCAAGCAGGTTAGGCTAGGTGGATGCGGTAGGTCCGAATATAAGGATGGAGACTTTGACATTCTTGCGGTTGTGGTTGACGAAGATGTGCTTATTTTCACATGGGACGAAGTAAAAGGTAAGACATCCATGTGTGTCGGCAAGAGAAACAAAGGCATAAAACTATAGgag                                                                <  1:576238/301‑1 (MQ=255)`  
                                                                                                                                                                                                                                              `cAAAGCTGGGTACACTGTCTATTACCCTATGCTGACTCAGAGTAAAGAGGACTTGGTTGTATGTAAGGATGGTAAATTTAGTAAGGTTCAGGTTAAAACAGCCACAACGGTTCAAACCAACACAGGAGATGCCAAGCAGGTTAGGCTAGGTGGATGCGGTAGGTCCGAATATAAGGATGGAGACTTTGACATTCTTGCGGTTGTGGTTGACGAAGATGTGCTTATTTTCACATGGGACGAAGTAAAAGGTAAGACATCCATGTGTGTCGGCAAGAGAAACAAAGGCATAAAACTATAGgag                                                                <  1:668158/301‑1 (MQ=255)`  
                                                                                                                                                                                                                                              `acaggCTGGGTACACTGTCTATTACCCTATGCTGACTCAGAGTAAAGAGGACTTGGTTGTATGTAAGGATGGTAAATTTAGTAAGGTGCAGGTTAAAACAGCCACAACGGTTCAAACCAACACAGGAGATGCCAAGCAGGTTAGTCTAGGTGGATGCGGTAGGTCCGAATATAAGGATGGATACTTTGACATTCTTGCGGTTGTGGTTGACGAAGATGTGCTTATTTTCACATGGGACGAAGTAAAAGGTAAGACATCCATGTGTGTCGGCAAGAGAAACAAAGGCATAAAACTATAGgag                                                                <  1:115636/297‑1 (MQ=255)`  
                                                                                                                                                                                                                                                   `cTGGGTACACTGTCTATTACCCTATGCTGACTCAGAGTAAAGAGGACTTGGTTGTATGgaaggattgatgacattcgtagtacgtacatcatttcgtgaggccattaaggacatgcacgaagagtatgcagataaggactctttcaaacaatctcctgcaacagtacgggaggtgttctgatgtctgactacctgaaagtgctgcaagcaatcaaaagttgccctaagactttccagtccaactatgtacggaacaatgcgagcctcgtagcggaggccgcttcccgtggtcacatctcgt                                                           >  1:274283‑M1/1‑58 (MQ=255)`  
                                                                                                                                                                                                                                                         `aacaggTCTATTACCCTATGCTGACTCAGAGTAAAGAGGACTTGGTTGTATGTAAGGATGGTAAATTTAGTAAGGTTCAGGTTAAAACAGCCACAACGGTTCAAACCAACACAGGAGATGCCAAGCAGGTTAGGCTAGGTGGATGCGGTAGGTCCGAATATAAGGATGGAGACTTTGACATTCTTGCGGTTGTGGTTGACGAAGATGTGCTTATTTTCACATGGGACGAAGTAAAAGGTAAGACATCCATGTGTGTCGGCAAGAGAAACAAAGGCATAAAACTATAGGAGAAATTATTATg                                                     <  1:183786/296‑1 (MQ=255)`  
                                                                                                                                                                                                                                                          `gaaaagcTATTACCCTAGGCTAACTCAAAGTAAAGAGGACTTGGTTGTATGTAAGGATGGTAAATTGAGTAAGGTTCAGGTTAAAACAGCCACAACGGTTCAAACCAACACAGGAGATGCCAAGCAGGTTAGGCTAGGGGGATGCGGTAGGTCCGAATATAAGGATGGAGACTTTGACATTCTTGCGGTTGTGGTTGACGAAGATGTGCTTATTTTCACATGGGACGAAGTAAAAGGTAAGACATCCATGTGTGTAGGCAAGAGAAACAAAGGCATAAAACTATAGGAGAAATTATTATgg                                                    <  1:364434/295‑1 (MQ=255)`  
                                                                                                                                                                                                                                                           `aaggTCTATTACCCTATGCTGACTCAGAGTAAAGAGGACTTGGTTGTATGTAAGGATGGTAAATTTAGTAAGGTTCAGGTTAAAACAGCCACAACGGTTCAAACCAACACAGGAGATGCCAAGCAGGTTAGGCGAGGTGGATGCGGTAGGTCCGAATATAAGGATGGAGACTTTGACATTCTTGCGGTTGTGGTTGACGAAGATGTGCTTATTTTCACATGGGACGAAGTAAAAGGTAAGACATCCATGTGTGTCGGCAAGAGAAACAAAGGCATAAAACTATAGGAGAAATTATTATGGc                                                   <  1:515041/298‑1 (MQ=255)`  
                                                                                                                                                                                                                                                            `cTGTCTATTACCCTATGCTGACTCAGAGTAAAGAGGACTTGGTTGTATGTAAGGATGGTAAATTTAGTAAGGTTCAGGTTAAAACAGCCACAACGGTTCAAACCAACACAGGAGATGCCAAGCAGGTTAGGCTAGGTGGATGCGGTAGGTCCGAATATAAGGATGGAGACTTTGACATTCTTGCGGTTGTGGTTGACGAAGATGTGCTTATTTTCACATGGGACGAAGTAAAAGGTAAGACATCCATGTGTGTCGGCAAGAGAAACAAAGGCATAAAAcctgtctcttatacacatctccg                                                  >  1:164111/1‑279 (MQ=255)`  
                                                                                                                                                                                                                                                             `tGTCTATTACCCTATGCTGACTCAGATTAAAGAGGAATTGGTTGTATGgaaggattgatgacattcgtagtacgtacatcatttcgtgaggccattaaggacatgcacgaagagtatgcagataaggactctttcaaacaatctcctgcaacagtacgggaggtgttctgatgtctgactacctgaaagtgatgcaagcaatcaaaagttgccctaagactttccagtccaactatgtacggaacaatgcgagcctcgtagcggaggccgcttcccgtggtcacatctcgtgcctgactac                                                 <  1:267058‑M1/301‑254 (MQ=255)`  
                                                                                                                                                                                                                                                              `gTCTATTACCCTATGCTGACTCAGAGTAAAGAGGACTTGGTTGTATGgaaggattgatgacattcgtagtacgtacatcatttcgtgaggccattaaggacatgcacgaagagtatgcagataaggactctttcaaacaatctcctgcaacagtacgggaggtgttctgatgtctgactacctgaaagtgctgcaagcaatcaaaagttgccctaagactttccagtccaactatgtacggaacaatgcgagcctcgtagcggaggccgcttcccgtggtcacatatcgtgcccggtctct                                                >  1:612512‑M1/1‑47 (MQ=255)`  
                                                                                                                                                                                                                                                              `gTCTATTACCCTATGCTGACTCAGAGTAAAGAGGACTTGGTTGTATGgaaggattgatgacattcgtagtacgtacatcatttcgtgaggccattaaggacatgcacgaagagtatgcagataaggactctttcaaacaatctcctgcaacagtacgggaggtgttctgatgtctgactacctgaaagtgctgcaagcaatcaaaagttgccctaagactttacagtccaactatgtacggaacaatgcgagcctcgtagcggaggccgcttcccgtggtcacatctcgtgcctgactact                                                >  1:100076‑M1/1‑47 (MQ=255)`  
                                                                                                                                                                                                                                                              `gTCTATTACCCTATGCTGACTCAGAGTAAAGAGGACTTGGTTGTATGTAAGGATGTTAAATTTAGTAAGGTTCAGGTTAAAACAGCCACAACGGTTCAAACCAACACAGGAGATGCCAAGCAGGTTAGGCTAGGTGGATGCGGTAGGTCCGAATATAAGGATGGAGACTTTGACATTCTTGCGGTTGTGGTTGACGAAGATGTGCTTATTTTCACATGGGACGAAGTAAAAGGTAAGACATCCATGTGTGTCGGCAAGAGAAACAAAGGCATAAAACTATAGGAGAAattctgtctcttat                                                >  1:604601/1‑290 (MQ=255)`  
                                                                                                                                                                                                                                                              `gTCTATTACCCTATGCTGACTCAGAGTAAAGAGGACTTGGTTGTATGTAAGGATGGTAAATTTAGTAAGGTTCAGGTTAAAACAGCCACAACGGTTCAAACCAACACAGGAGATGCCAAGCAGGTTAGGCTAGGTGGATGCGGTAGGTCCGAATATAAGGATGGAGACTTTGACATTCTTGCGGTTGTGGTTGACGAAGATGTGCTTATTTTCACATGGGACGAAGTAAAAGGTAAGACATCCATGTGTGTCGGCAAGAGAAACAAAGGCATAAAACTATAGGAGACATTATTATGGCTAt                                                >  1:660434/1‑301 (MQ=255)`  
                                                                                                                                                                                                                                                              `gTCTATTACCCTATGCTGACTCAGAGTAAAGAGGACTTGGTTGTATGTAAGGATGGTAAATTTAGTAAGGTTCAGGTTAAAACAGCCACAACGGTTCAAACCAACACAGGAGATGCCAAGCAGGTTAGGCTAGGTGGATGCGGTAGGTCCGAATATAAGGATGGAGACTTTGACATTCTTGCGGTTGTGGTTGACGAAGATGTGCTTATTTTCACATGGGACGAAGTAAAAGGTAAGACATCCATGTGTGTCGGCAAGAGAAACAAAGGCATAAAACTATAGGAGAAATTATTATGGCTAt                                                >  1:438379/1‑301 (MQ=255)`  
                                                                                                                                                                                                                                                              `gTCTATTACCCTATGCTGACTCAGAGTAAAGAGGACTTGGTTGTATGTAAGGATGGTAAATTTAGTAAGGTTCAGGTTAAAACAGCCACAACGGTTCAAACCAACACAGGAGATGCCAAGCAGGTTAGGCTAGGTGGATGCGGTAGGTCCGAATATAAGGATGGAGACTTTGACATTCTTGCGGTTGTGGTTGACGAAGATGTGCTTATTTTCACATGGGACGAAGTAAAAGGTAAGACATCCATGTGTGTCGGCAAGAGAAACAAAGGCATAAAACTATAGGAGAAATTATTATAGCTAt                                                >  1:770809/1‑301 (MQ=255)`  
                                                                                                                                                                                                                                                                `ttaTTACCCTATGCTGACTCAGAGTAAAGAGGACTTGGTTGTATGgaaggattgatgacattcgtagtacgtacatcatttcgtgaggccattaaggacatgcacgaagagtatgcagataaggactctttcaaacaatctcctgcaacagtacgggaggtgttctgatgtctgactacctgaaagtgctgcaagcaatcaaaagttgccctaagactttccagtccaactatgtacggaacaatgcgagcctcgtagcggaggccgcttcccgtggtcacatctcgtgcctgactactag                                              <  1:619477‑M1/300‑257 (MQ=255)`  
                                                                                                                                                                                                                                                                `cTATTACCCTATGCTGACTCAGAGTAAAGAGGACTTGGTTGTATGgaaggattgatgacattcgtagtacgtacatcatttcgtgaggccattaaggacatgcacgaagagtatgcagataaggactctttcaaacaatctcctgcaacagtacgggaggtgttctgatgtctgactacctgaaagtgctgcaagcaatcaaaagttgccctaagactttccagtccaactatgtacggaacaatgcgagcctcgtagcggaggccgcttcccgtggtcacatctcgtgcctgactactag                                              >  1:211398‑M1/1‑45 (MQ=255)`  
                                                                                                                                                                                                                                                                `cTATTACCCTATGCTGACTCAGAGTAAAGAGGACTTGGTTGTATGTAAGGATGGTAAATTTAGTAAGGTTCAGGTTATAACAGCCACAACGGTTCAAACCAACACAGGAGATGCCAAGCAGGTTAGGCTAGGTGGATGCGGTAGGTCCGAATATAAGGATGGAGACTTTGACATTCTTGCGGTTGTGGTTGACGAAGATGTGCTTATTTTCACATGGGACGAAGTAAAAGGTAAGACATCCATGTGTGTCGGCAAGAGAAACAAAGGCATAAAACTATATGCGAAATTATTATGGCTCTgg                                              >  1:296006/1‑300 (MQ=255)`  
                                                                                                                                                                                                                                                                 `tATTACCCTATGCTGACTCAGAGTAAAGAGGACTTGGTTGTATGTAAGGATGGTAAATTTAGTAAGGTTCAGGTTAAAACAGCCACAACGGTTCAAACCAACACAGGAGATGCCAAGCAGGTTAGGCTAGGTGGATGCGGTAGGTCCGAATATAAGGATGGAGACTTTGACATTCTTGCGGTTGTGGTTGACGAAGATGTGCTTATTTTCACATGGGACGAAGTAAAAGGTAAGACATCCATGTGTGTCGGCAAGAGAAACAAAGGCATAAAACTATAGGAGAAATTATTATGGCTATGAc                                             <  1:121995/301‑1 (MQ=255)`  
                                                                                                                                                                                                                                                                  `agagacagatgctgactCAGAGTAAAGAGGACTTGGTTGTATGgaaggattgatgacattcgtagtacgtacatcatttcgtgaggccattaaggacatgcacgaagagtatgcagataaggactctttcaaacaatctcctgcaacagtacgggaggtgttctgatgtctgactacctgaaagtgctgcaagcaatcaaaagttgccctaagactttccagtccaactatgtacggaacaatgcgagcctcgtagcggaggccgcttcccgtggtcacatctcgtgcctgactactagtg                                            <  1:675873‑M1/293‑259 (MQ=255)`  
                                                                                                                                                                                                                                                                    `tACCCTATGCTGACTCAGAGTAAAGAGGACTTGGTTGTATGgaaggattgatgacattcgtagtacgtacatcatttcgtgaggccattaaggacatgcacgaagagtatgcagataaggactctttcaaacaatctcctgcaacagtacgggaggtgttctgatgtctgactacctgaaagtgctgcaagcaatcaaaagttgccctaagactttccagtccaactatgtacggaacaatgcgagcctcgtagcggaggccgcttcccgtggtcacatctcgtgcctgactactagtgga                                          <  1:491843‑M1/301‑261 (MQ=255)`  
                                                                                                                                                                                                                                                                     `aCCCTATGCTGACTCAGAGTAAAGAGGACTTGGTTGTATGgaaggattgatgacattcgtagtacgtacatcatttcgtgaggcaattaaggacatgcactaagagtatgcagataaggactctttcaaacaatctcctgcaacagtacgggatgtgttctgatgtctgactaactgaaagtgctgcaagcaatcacaagttgccctaatactttccattccaactatatacggaacaatgcgagactcgtagcgtaggccgcttcccgttgtcacatatcttgcctgactactagtggtc                                         >  1:63310‑M1/1‑40 (MQ=255)`  
                                                                                                                                                                                                                                                                     `aCCCTATGCTGACTCAGAGTAAAGAGGACTTGGTTGTATGTAAGGATGGTAAATTTAGTAAGGTTCAGGTTAAAACAGCCACAACGGTTCAAACCAACACAGGAGATGCCAAGCAGGTTAGGCTAGGTGGATGCGGTAGGTCCGAATATAAGGATTGAGACTTTGACATTCTTGCGGTTGTGGTTGACGAAGATGTGCTTATTTTCACATGGGACGAAGTAAAAGGTAAGACATCCATGTGTGTCGGCAAGAGAAACAAAGGCATAAAACTATAGGAGAAATTATTATGGCTATGAcctgt                                         >  1:639468/1‑297 (MQ=255)`  
                                                                                                                                                                                                                                                                     `aCCCTATGCTGACTCAGAGTAAAGAGGACTTGGTTGTATGTAAGGATGGTAAATTTAGTAAGGTTCAGGTTAAAACAGCCACAACGGTTCAAACCAACACAGGAGATGCCAAGCAGGTTAGGCTAGGTGGATGCGGTAGGTCCGAATATAAGGATGGAGACTTTGACATTCTTGCGGTTGTTGTTGACGAAGATGTGCTTATTTTCACATGGGACGAAGTAAAAGGTAAGACATCCATGTGTGTCGGCAAGAGAAACAAAGGCATAAAACTATAGGAGaactgtctcttatacacatctcc                                         >  1:597309/1‑280 (MQ=255)`  
                                                                                                                                                                                                                                                                     `aCCCTATGCTGACTCAGAGTAAAGAGGACTTGGTTGTATGTAAGGATGGTAAATTTAGTAAGGTTCAGGTTAAAACAGCCACAACGGTTCAAACCAACACAGGAGATGCCAAGCAGGTTAGGCTAGGTGGATGCGGTAGGTCCGAATATAAGGATGGAGACTTTGACATTCTTGCGGTTGTGGTTGACGAAGATGTGCTTATTTTCACATGGGACGAAGTAAAAGGTAAGACATCCATGTGTGTCGGCAAGAGAAACAAAGGCATAAAACTATAGGAGAAATTATTATGGCtgtctcttat                                         >  1:228549/1‑292 (MQ=255)`  
                                                                                                                                                                                                                                                                     `aCCCTATGCTGACTCAGAGTAAAGAGGACTTGGTTGTATGTAAGGATGGTAAATTTAGTAAGGTTCAGGTTAAAACAGCCACAACGGTTCAAACCAACACAGGAGATGCCAAGCAGGTTAGGCTAGGTGGATGCGGTAGGTCCGAATATAAGGATGGAGACTTTGACATTCTTGCGGTTGTGGTTGACGAAGATGTGCTTATTTTCACATGGGACGAAGTAAAAGGTAAGACATCCATGTGTGTCGGCAAGAGAAACAAAGGCATAAAACTATAGGAGAAATTATTATGGCTATGACAAAg                                         >  1:322354/1‑301 (MQ=255)`  
                                                                                                                                                                                                                                                                       `agagacaggACTAAGAGTAAAGAGGACTTGGTTGTATGTAAGGATGGTAAATTTAGTAAGGTTCAGGTTAAAACAGCCACAACGGTTCAAACCAACACAGGAGATTCCAAGCAGGTTATGCTAGGTGGATGCGGTAGGTCCGAATATAAGGATGGAGACTTTGACATTCTTGCGGTTGTGGTTGACGAAGATGTGCTTATTTTCACATGGGACGAAGTAAAAGGTAAGACATCCATGTGTGTCGGCAAGAGAAACAAAGGCATAAAACTATAGGAGAAATTATTATGGCTATGACAAAGaa                                       <  1:816033/293‑1 (MQ=255)`  
                                                                                                                                                                                                                                                                             `cTGACACAGAATAAAGAGGACTTGGTTGTAGGAAAGGATGGTAAATTTAGTAAGGTTCAGGTTAAAACAGCCACAACGGTTCAAACCAACACAGGGGATGCCAAGCAGGTTAGGCTAGGTGGATGCGGTAGGTCCGAATATAAGGATGGAGACTTTAACATTCTTGCGGTTGTGGTTGACGAAGATGTGCTTATTTTCACATGGGACGAAGTAAAAGGTAAGACATCAATGTGTGTCGGCAAGAGAAACAAAGGCATAAAACTATAGGAGAAATTATTATGGCTATGACAAAGAAATTTaa                                 <  1:424982/301‑1 (MQ=255)`  
                                                                                                                                                                                                                                                                                    `agagTAAAGAGGACTTGGTTGTATGTAAGGATGGTAAATTTAGTAAGGTTCAGGTTAAAACAGCCACAACGGTTCAAACCAACACAGGAGATGCCAAGCAGGTTAGGCTAGGTGGATGCGGTAGGTCCGAATATAAGGATGGAGACTTTGACATTCTTGCGGTTGTGGTTGACGAAGATGTGCTTATTTTCACATGGGACGAAGTAAAAGGTAAGACATCCATGTGTGTCGGCAAGAGAAACAAAGGCATAAAACTATAGGAGAAATTATTATGGCTATGACAAAGAAATTTAAAgtctgt                          >  1:484504/1‑297 (MQ=255)`  
                                                                                                                                                                                                                                                                                     `gagTAAAGAGGACTTGGTTGTATGgaaggattgatgacattcgtagtacgtacatcatttcgtgaggccattaaggacatgcacgaagagtatgcagataaggactctttcaaacaatctactgcaacagtacgggaggtgttctgatgtctgactacctgaaagtgctgcaagcaatcaaaagttgccctaagactttccagtccaactatgtacggaacaatgcgagcctcgtagcggaggccgcttcccgtggtcacatctcgtgcctgactactagtggacgtaacggtggcgcttg                         >  1:755782‑M1/1‑24 (MQ=255)`  
                                                                                                                                                                                                                                                                                                 `cTTGGTTGTATGTAAGGATGGTAAATTTAGTAAGGTTCAGGTTAAAACAGCCACAACGGTTCAAACCAACACAGGAGATGCCAAGCAGGTTAGGCTAGGTGGATGCGGTAGGTCCGAATATAAGGATGGAGACTTTGACATTCTTGCGGTTGTGGTTGACGAAGATGTGCTTATTTTCACATGGGACGAAGTAAAAGGTAAGACATCCATGTGTGTCGGCAAGAGAAACAAAGGCATAAAACTATAGGAGAAATTATTATGGCTATGACAAAGAAATTTAAAGTGTCCtctgtctcttata             >  1:685221/1‑289 (MQ=255)`  
                                                                                                                                                                                                                                                                                                    `ggTTGTATGTAAGGATGGTAAATTTAGTAAGGTTCAGGTTAAAACAGCCACAACGGTTCAAACCAACACAGGAGATGCCAAGCAGGTTAGGCTAGGTGGATGCGGTAGGTCCGAATATAAGGATGGAGACTTTGACATTCTTGCGGTTGTGGTTGACGAAGATGTGCTTATTTTCACATGGGACGAAGTAAAAGGTAAGACATCCATGTGTGTCGGCAAGAGAAACAAAGGCATAAAACTATAGGAGAAATTATTATGGCTATGACAAAGAAATTTAAAGTGTCCTTCGACGTTACCGCaa          >  1:447247/1‑301 (MQ=255)`  
                                                                                                                                                                                                                                                                                                       `tgtatgtaAGGATGGTAAATTTAGTAAGGTTCAGGTTAAAACAGCCACAACGGTTCAAACCAACACAGGAGATGCCAAGCAGGTTAGGCTAGGTGGATGCGGTAGGTCCGAATATAAGGATGGAGACTTTGACATTCTTGCGGTTGTGGTTGACGAAGATGTGCTTATTTTCACATGGGACGAAGTAAAAGGTAAGACATCCATGTGTGTCGGCAAGAGAAACAAAGGCATAAAACTATAGGAGAAATTATTATGGCTATGACAAAGAAATTTAAAGTGTCCTTCGACGTTACCGCAAAGa       <  1:776337/301‑1 (MQ=255)`  
                                                                                                                                                                                                                                                                                                        `gtatgtaAGGATGGTAAATTTAGTAAGGTTCAGGTTAAAACAGCCACAACGGTTCAAACCAACACAGGAGATGCCAAGCAGGTTAGGCTAGGTGGATGCGGTAGGTCCGAATATAAGGATGGAGACTTTGACATTCTTGCGGTTGTGGTTGACGAAGATGTGCTTATTTTCACATGGGACGAAGTAAAAGGTAAGACATCCATGTGTGTCGGCAAGAGAAACAAAGGCATAAAACTATAGGAGAAATTATTATGGCTATGACAAAGAAATTTAAAGTGTCCTTCGACGTTACCGCAAAGAt      >  1:191172/1‑301 (MQ=255)`  
                                                                                                                                                                                                                                                                                                        `gTATGgaaggattgatgacattcgtagtacgtacatcatttcgtgaggccattaaggacatgcacgaagagtatgcagataaggactctttcaaacaatctcctgcaacagtacgggaggtgttctgatgtctgactacctgaaagtgctgcaagcaatcaaaagttgccctaagactttccagtccaactatgtacggaacaatgcgagcctcgtagcggaggccgcttcccgtggtcacatctcgtgcctgactactagtggacgtaacggtggggcttgggaaatcactgcttccggt      >  1:589769‑M1/1‑5 (MQ=255)`  
                                                                                                                                                                                                                                                                                                         `tATGgaaggattgatgacattcgtagtacgtacatcatttcgtgaggccattaaggacatgcacgaagagtatgcagataaggactctttcaaacaatctcctgcaacagtacgggagttgttctgatgtctgactacctgaaagtgctgcaagcaatcaaaagttgccctaagactttccagtccaactatgtacggaacaatgcgagcctcgtagcggaggccgcttcccgtggtcacatctcgtgcctgactactagtggacgtaacggtggcgcttgggaaatcactgcttccggta     <  1:337638‑M1/301‑298 (MQ=255)`  
                                                                                                                                                                                                                                                                                                          `atgtaAGGATGGTAAATTTAGTAAGGTTCAGGTTAAAACAGCCACAACGGTTCAAACCAACACAGGAGATGCCAAGCAGGTTAGGCTAGGTGGATGCGGTAGGTCCGAATATAAGGATGGAGACTTTGACATTCTTGCGGTTGTGGTTGACGAAGATGTGCTTATTTTCACATGGGACGAAGTAAAAGGTAAGACATCCATGTGTGTCGGCAAGAGAAACAAAGGCATAAAACTATAGGAGAAATTATTATGGCTATGACAAAGAAATTTAAAGTGTCCTTCGATGTTACCGCAAAGATgt    >  1:380860/1‑301 (MQ=255)`  

                                                                                                                                                                                                                                                                                                            `|`                                                                                                                                                                                                                                                                                                              
`GGGTACATGATGAAATCCAAGTAGGCTGCCGTACCGAAGAGATTGCTCAGGTGGTCATTGAGACCGCACAAGAAGCGATGCGCTGGGTTGGAGACCACTGGAACTTCCGGTGTCTTCTGGATACCGAAGGTAAGATGGGTCCTAATTGGGCGATTTGCCACTGATACAGGAGGCTACTCATGAACGAAAGACACTTAACAGGTGCTGCTTCTGAAATGCTAGTAGCCTACAAATTTACCAAAGCTGGGTACACTGTCTATTACCCTATGCTGACTCAGAGTAAAGAGGACTTGGTTGTATGTAAGGATGGTAAATTTAGTAAGGTTCAGGTTAAAACAGCCACAACGGTTCAAACCAACACAGGAGATGCCAAGCAGGTTAGGCTAGGTGGATGCGGTAGGTCCGAATATAAGGATGGAGACTTTGACATTCTTGCGGTTGTGGTTGACGAAGATGTGCTTATTTTCACATGGGACGAAGTAAAAGGTAAGACATCCATGTGTGTCGGCAAGAGAAACAAAGGCATAAAACTATAGGAGAAATTATTATGGCTATGACAAAGAAATTTAAAGTGTCCTTCGACGTTACCGCAAAGATGTCG  >  NC_001604.1/16304‑16904`

  

Alignment Legend

Aligned base mismatch/match (shaded by quality score): `ATCG/ATCG` `< 3 ≤` `ATCG/ATCG` `< 11 ≤` `ATCG/ATCG` `< 27 ≤` `ATCG/ATCG` `< 37 ≤` `ATCG/ATCG` `< 38 ≤` `ATCG/ATCG`

Unaligned base: `atcg`    Masked matching base: `atcg`    Alignment gap: `‑`    Deleted base: `‑`

Reads not counted as support for junction

read\_name Not counted due to insufficient overlap past the breakpoint.

read\_name Not counted due to not crossing MOB target site duplication.
